# 12-Chloro-6-cyclo­hexyl-5,6,7,12-tetra­hydro­dibenzo[*c*,*f*][1,5]aza­stibocine

**DOI:** 10.1107/S1600536811021477

**Published:** 2011-06-11

**Authors:** Weiguo Yi, Nianyuan Tan

**Affiliations:** a Hunan Chemical Vocational Technology College, Zhuzhou 421000, People’s Republic of China; b College of Chemistry and Chemical Engineering, Hunan Institute of Engineering, Xiangtan 411104, People’s Republic of China

## Abstract

In the title organometallic complex, [Sb(C_20_H_23_N)Cl], the central anti­mony-containing part of the complex exhibits a pseudo-trigonal-bipyramidal geometry, where two C atoms and a lone electron pair of the Sb atom exist at the equatorial positions, while the N and Cl atoms are located at the apical positions, and a transannular inter­action exists between the Sb and N atoms on 1,5-aza­stibocine. Inter­molecular C—H⋯Cl hydrogen bonds are also observed.

## Related literature

For general background, see: Yin *et al.* (2008 ▶); Chovancová *et al.* (2009 ▶); Opris *et al.* (2009 ▶); Svoboda *et al.* (2010 ▶); Tan & Zhang (2011 ▶). For related structures, see: Kakusawa *et al.* (2006 ▶); Xia *et al.* (2010 ▶).
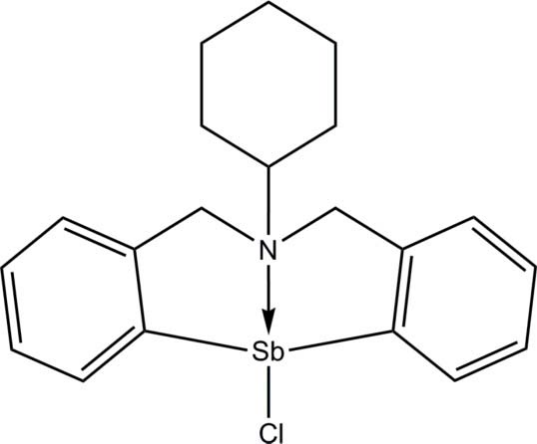


## Experimental

### 

#### Crystal data

[Sb(C_20_H_23_N)Cl]
*M* *_r_* = 434.59
Monoclinic, 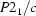

*a* = 10.0771 (7) Å
*b* = 16.2881 (12) Å
*c* = 12.2040 (9) Å
β = 111.812 (1)°
*V* = 1859.7 (2) Å^3^
*Z* = 4
Mo *K*α radiation
μ = 1.63 mm^−1^
*T* = 293 K
0.37 × 0.35 × 0.21 mm

#### Data collection

Bruker SMART CCD area-detector diffractometer
Absorption correction: multi-scan (*SADABS*; Sheldrick, 1999 ▶) *T*_min_ = 0.653, *T*_max_ = 1.000
10058 measured reflections
3644 independent reflections
3107 reflections with *I* > 2σ(*I*)
*R*_int_ = 0.047

#### Refinement

*R*[*F* ^2^ > 2σ(*F* ^2^)] = 0.032
*wR*(*F* ^2^) = 0.092
*S* = 1.05
3644 reflections
209 parameters
H-atom parameters constrained
Δρ_max_ = 0.78 e Å^−3^
Δρ_min_ = −0.55 e Å^−3^

### 

Data collection: *SMART* (Bruker, 1997 ▶); cell refinement: *SAINT* (Bruker, 1997 ▶); data reduction: *SAINT*; program(s) used to solve structure: *SHELXS97* (Sheldrick, 2008 ▶); program(s) used to refine structure: *SHELXL97* (Sheldrick, 2008 ▶); molecular graphics: *SHELXTL* (Sheldrick, 2008 ▶); software used to prepare material for publication: *SHELXL97*.

## Supplementary Material

Crystal structure: contains datablock(s) I, global. DOI: 10.1107/S1600536811021477/vm2099sup1.cif

Structure factors: contains datablock(s) I. DOI: 10.1107/S1600536811021477/vm2099Isup2.hkl

Supplementary material file. DOI: 10.1107/S1600536811021477/vm2099Isup3.cml

Additional supplementary materials:  crystallographic information; 3D view; checkCIF report

## Figures and Tables

**Table 1:** Hydrogen-bond geometry (Å, °)

*D*—H⋯*A*	*D*—H	H⋯*A*	*D*⋯*A*	*D*—H⋯*A*
C7—H7*A*⋯Cl1^i^	0.97	2.80	3.695 (4)	154

Symmetry code: (i) 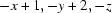.

**Fig. 1.**
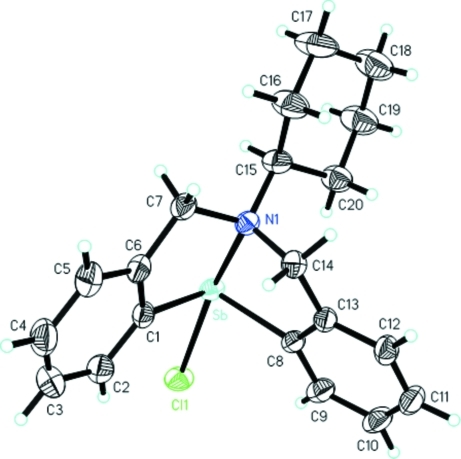
The molecular structure of the title compound with atomic numbering scheme. Displacement ellipsoids are drawn at the 30% probability level.

**Fig. 2.**
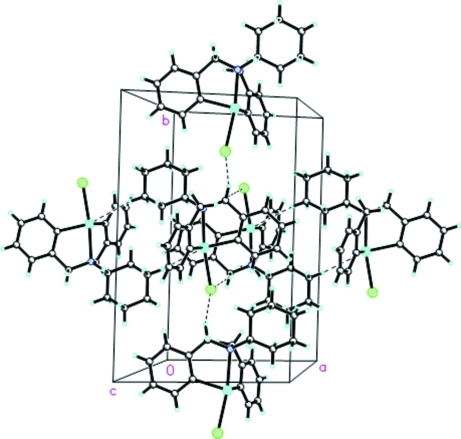
A packing diagram of the title compound viewed down the a axis.

## supplementary crystallographic information

### Comment

The chemistry of hypervalent compounds bearing heavier pnictogens (in particular Sb, Bi) has been studied intensively in recent years (Yin *et al.*, 2008; Chovancová *et al.*, 2009; Svoboda *et al.*, 2010; Tan & Zhang, 2011). Intramolecular interactions between antimony and *sp*^3^-nitrogen atoms have been widely reported (Kakusawa *et al.*, 2006; Opris *et al.*, 2009; Xia *et al.*, 2010). Here, we reported the crystal structure of the title organometallic complex (Fig. 1). The central antimony-containing part of the complex shows a distorted pseudo trigonal-bipyramidal structure. The C1, C8 atoms along with a lone electron pair of the Sb atom exist at the equatorial positions while the N1 and Cl1 atoms are located at the apical positions. The Sb–C1 and Sb–C8 distance is 2.144 (4) Å and 2.134 (3) Å, respectively. The C1–Sb–C8 angle is 98.17 (12)°, while the N1–Sb–Cl1 angle is 162.92 (7)° (rather than 180°). The Sb–N1 distance (2.397 (3) Å) is shorter than the sum of the van der Waals radii of nitrogen and antimony atoms (3.74 Å) (Kakusawa *et al.*, 2006), indicating that coordination exists between the two atoms. The complex also displays intermolecular hydrogen-bonding interaction between the CH_2_ groups and chlorine atom Cl1 (Table 1).

### Experimental

*N*,*N*-bis(2-bromobenzyl)cyclohexanamine (2.186 g, 5.0 mmol) was allowed to react with n-BuLi (2.5 *M*, 4.0 ml, 10 mmol) at -50 *^o^*C, and the resulting solution was added to a mixture of SbCl_3_ (1.141 g, 5.0 mmol) in Et_2_O (80 ml) at -78 *^o^*C. The obtained mixture was gradually warmed to room temperature and stirred for 12 h. Then the solvent was removed under vacuum and the residue was extracted with toluene, and the insoluble material was removed by filtration. The organic layer was washed with de-ionized H_2_O and dried over anhydrous Na_2_SO_4_. After the solvent was removed under reduced pressure, the residue was recrystallized from CH_2_Cl_2_/hexane to obtain the title compound in the form of colorless crystals.

### Refinement

All H atoms were positioned geometrically and refined using a riding model, with C—H = 0.93 Å for aryl, 0.98 Å for methine and 0.97 Å for methylene H atoms, respectively. *U*_iso_(H)= 1.2*U*_eq_(C) for all H atoms.

### Crystal data

**Table:**

[Sb(C_20_H_23_N)Cl]	*F*(000) = 872
*M**_r_* = 434.59	*D*_x_ = 1.552 Mg m^−^^3^
Monoclinic, *P*2_1_/*c*	Melting point: 527.15 K
Hall symbol: -P 2ybc	Mo *K*α radiation, λ = 0.71073 Å
*a* = 10.0771 (7) Å	Cell parameters from 5285 reflections
*b* = 16.2881 (12) Å	θ = 4.4–55.7°
*c* = 12.2040 (9) Å	µ = 1.63 mm^−^^1^
β = 111.812 (1)°	*T* = 293 K
*V* = 1859.7 (2) Å^3^	Prismatic, colorless
*Z* = 4	0.37 × 0.35 × 0.21 mm

### Data collection

**Table:**

Bruker SMART CCD area-detector diffractometer	3644 independent reflections
Radiation source: fine-focus sealed tube	3107 reflections with *I* > 2σ(*I*)
graphite	*R*_int_ = 0.047
Detector resolution: 10.00 pixels mm^-1^	θ_max_ = 26.0°, θ_min_ = 2.2°
φ and ω scans	*h* = −12→7
Absorption correction: multi-scan (*SADABS*; Sheldrick, 1999)	*k* = −20→19
*T*_min_ = 0.653, *T*_max_ = 1.000	*l* = −15→14
10058 measured reflections	

### Refinement

**Table:**

Refinement on *F*^2^	Secondary atom site location: difference Fourier map
Least-squares matrix: full	Hydrogen site location: inferred from neighbouring sites
*R*[*F*^2^ > 2σ(*F*^2^)] = 0.032	H-atom parameters constrained
*wR*(*F*^2^) = 0.092	*w* = 1/[σ^2^(*F*_o_^2^) + (0.0544*P*)^2^ + 0.1065*P*] where *P* = (*F*_o_^2^ + 2*F*_c_^2^)/3
*S* = 1.05	(Δ/σ)_max_ = 0.020
3644 reflections	Δρ_max_ = 0.78 e Å^−^^3^
209 parameters	Δρ_min_ = −0.55 e Å^−^^3^
0 restraints	Extinction correction: *SHELXL97* (Sheldrick, 2008), Fc^*^=kFc[1+0.001xFc^2^λ^3^/sin(2θ)]^-1/4^
Primary atom site location: structure-invariant direct methods	Extinction coefficient: 0.0030 (4)

### Special details

**Table:**

Geometry. All e.s.d.'s (except the e.s.d. in the dihedral angle between two l.s. planes) are estimated using the full covariance matrix. The cell e.s.d.'s are taken into account individually in the estimation of e.s.d.'s in distances, angles and torsion angles; correlations between e.s.d.'s in cell parameters are only used when they are defined by crystal symmetry. An approximate (isotropic) treatment of cell e.s.d.'s is used for estimating e.s.d.'s involving l.s. planes.
Refinement. Refinement of *F*^2^ against ALL reflections. The weighted *R*-factor *wR* and goodness of fit *S* are based on *F*^2^, conventional *R*-factors *R* are based on *F*, with *F* set to zero for negative *F*^2^. The threshold expression of *F*^2^ > σ(*F*^2^) is used only for calculating *R*-factors(gt) *etc*. and is not relevant to the choice of reflections for refinement. *R*-factors based on *F*^2^ are statistically about twice as large as those based on *F*, and *R*- factors based on ALL data will be even larger.

### Fractional atomic coordinates and isotropic or equivalent isotropic displacement parameters (Å^2^)

**Table:**

	*x*	*y*	*z*	*U*_iso_*/*U*_eq_	
Sb	0.38578 (2)	1.037779 (13)	0.117005 (18)	0.04278 (12)	
Cl1	0.43763 (12)	1.19188 (5)	0.12783 (8)	0.0597 (3)	
N1	0.3869 (3)	0.89526 (15)	0.1662 (2)	0.0400 (6)	
C1	0.6082 (4)	1.0063 (2)	0.1912 (3)	0.0459 (8)	
C2	0.7215 (5)	1.0616 (3)	0.2281 (4)	0.0615 (10)	
H2	0.7033	1.1177	0.2246	0.074*	
C3	0.8613 (5)	1.0335 (3)	0.2700 (5)	0.0772 (14)	
H3	0.9367	1.0707	0.2947	0.093*	
C4	0.8882 (5)	0.9511 (3)	0.2750 (5)	0.0734 (13)	
H4	0.9822	0.9324	0.3034	0.088*	
C5	0.7772 (4)	0.8953 (3)	0.2383 (4)	0.0647 (11)	
H5	0.7968	0.8393	0.2424	0.078*	
C6	0.6365 (4)	0.9224 (2)	0.1953 (3)	0.0475 (8)	
C7	0.5159 (4)	0.8623 (2)	0.1497 (3)	0.0505 (9)	
H7A	0.4943	0.8521	0.0666	0.061*	
H7B	0.5436	0.8107	0.1917	0.061*	
C8	0.3459 (3)	1.04120 (18)	0.2769 (3)	0.0405 (7)	
C9	0.3038 (4)	1.1103 (2)	0.3214 (3)	0.0544 (9)	
H9	0.2980	1.1605	0.2836	0.065*	
C10	0.2703 (4)	1.1057 (3)	0.4213 (4)	0.0662 (11)	
H10	0.2449	1.1530	0.4515	0.079*	
C11	0.2746 (4)	1.0317 (3)	0.4757 (4)	0.0641 (11)	
H11	0.2484	1.0286	0.5410	0.077*	
C12	0.3175 (4)	0.9621 (2)	0.4344 (3)	0.0530 (10)	
H12	0.3233	0.9124	0.4733	0.064*	
C13	0.3522 (3)	0.96613 (19)	0.3339 (3)	0.0413 (7)	
C14	0.4045 (4)	0.89040 (19)	0.2921 (3)	0.0445 (7)	
H14A	0.5048	0.8823	0.3398	0.053*	
H14B	0.3523	0.8431	0.3033	0.053*	
C15	0.2520 (4)	0.8556 (2)	0.0804 (3)	0.0546 (9)	
H15	0.2451	0.8713	0.0009	0.066*	
C16	0.2535 (5)	0.7640 (2)	0.0841 (5)	0.0772 (13)	
H16A	0.2598	0.7455	0.1615	0.093*	
H16B	0.3362	0.7435	0.0702	0.093*	
C17	0.1141 (5)	0.7300 (3)	−0.0122 (5)	0.1008 (18)	
H17A	0.1129	0.7441	−0.0898	0.121*	
H17B	0.1127	0.6706	−0.0065	0.121*	
C18	−0.0157 (5)	0.7648 (3)	0.0023 (5)	0.0968 (17)	
H18A	−0.1006	0.7448	−0.0607	0.116*	
H18B	−0.0193	0.7461	0.0767	0.116*	
C19	−0.0150 (5)	0.8545 (3)	0.0004 (5)	0.0885 (15)	
H19A	−0.0989	0.8749	0.0125	0.106*	
H19B	−0.0197	0.8731	−0.0765	0.106*	
C20	0.1202 (4)	0.8898 (2)	0.0964 (4)	0.0636 (10)	
H20A	0.1197	0.9492	0.0907	0.076*	
H20B	0.1217	0.8751	0.1739	0.076*	

### Atomic displacement parameters (Å^2^)

**Table:**

	*U*^11^	*U*^22^	*U*^33^	*U*^12^	*U*^13^	*U*^23^
Sb	0.05674 (19)	0.03541 (16)	0.03702 (16)	0.00115 (9)	0.01840 (11)	−0.00007 (8)
Cl1	0.0872 (7)	0.0321 (4)	0.0647 (6)	−0.0061 (4)	0.0338 (5)	−0.0033 (4)
N1	0.0432 (15)	0.0373 (14)	0.0405 (14)	0.0032 (11)	0.0168 (11)	−0.0007 (11)
C1	0.051 (2)	0.0490 (19)	0.0467 (19)	−0.0022 (16)	0.0285 (16)	−0.0007 (15)
C2	0.067 (3)	0.059 (2)	0.067 (3)	−0.007 (2)	0.035 (2)	−0.007 (2)
C3	0.057 (3)	0.096 (4)	0.084 (3)	−0.022 (2)	0.032 (2)	−0.017 (3)
C4	0.051 (2)	0.095 (4)	0.081 (3)	0.005 (2)	0.034 (2)	−0.002 (3)
C5	0.058 (2)	0.075 (3)	0.069 (3)	0.009 (2)	0.033 (2)	0.001 (2)
C6	0.053 (2)	0.050 (2)	0.048 (2)	0.0037 (16)	0.0287 (16)	0.0017 (15)
C7	0.061 (2)	0.0412 (18)	0.059 (2)	0.0029 (16)	0.0332 (18)	−0.0030 (16)
C8	0.0390 (17)	0.0445 (18)	0.0372 (17)	0.0020 (13)	0.0134 (13)	−0.0059 (13)
C9	0.053 (2)	0.058 (2)	0.050 (2)	0.0065 (17)	0.0155 (16)	−0.0105 (16)
C10	0.056 (2)	0.084 (3)	0.061 (3)	0.010 (2)	0.0237 (19)	−0.026 (2)
C11	0.056 (2)	0.094 (3)	0.049 (2)	−0.003 (2)	0.0279 (19)	−0.015 (2)
C12	0.044 (2)	0.076 (3)	0.040 (2)	−0.0035 (16)	0.0162 (16)	0.0037 (16)
C13	0.0347 (17)	0.052 (2)	0.0355 (17)	−0.0007 (13)	0.0118 (13)	−0.0021 (13)
C14	0.0472 (18)	0.0436 (18)	0.0433 (18)	0.0026 (14)	0.0177 (14)	0.0068 (14)
C15	0.057 (2)	0.0428 (19)	0.059 (2)	−0.0008 (16)	0.0163 (18)	−0.0080 (16)
C16	0.069 (3)	0.048 (2)	0.105 (4)	−0.0050 (19)	0.020 (2)	−0.012 (2)
C17	0.085 (4)	0.065 (3)	0.131 (5)	−0.017 (3)	0.016 (3)	−0.041 (3)
C18	0.066 (3)	0.080 (3)	0.128 (5)	−0.020 (3)	0.018 (3)	−0.021 (3)
C19	0.062 (3)	0.076 (3)	0.106 (4)	−0.004 (2)	0.007 (3)	−0.016 (3)
C20	0.051 (2)	0.057 (2)	0.075 (3)	−0.0014 (18)	0.0144 (19)	−0.0096 (19)

### Geometric parameters (Å, °)

**Table:**

Sb—C8	2.134 (3)	C10—H10	0.9300
Sb—C1	2.144 (4)	C11—C12	1.374 (5)
Sb—N1	2.397 (3)	C11—H11	0.9300
Sb—Cl1	2.5573 (9)	C12—C13	1.396 (5)
N1—C14	1.481 (4)	C12—H12	0.9300
N1—C7	1.487 (4)	C13—C14	1.503 (4)
N1—C15	1.518 (4)	C14—H14A	0.9700
C1—C2	1.391 (5)	C14—H14B	0.9700
C1—C6	1.393 (5)	C15—C16	1.493 (5)
C2—C3	1.385 (6)	C15—C20	1.518 (5)
C2—H2	0.9300	C15—H15	0.9800
C3—C4	1.367 (6)	C16—C17	1.560 (6)
C3—H3	0.9300	C16—H16A	0.9700
C4—C5	1.381 (6)	C16—H16B	0.9700
C4—H4	0.9300	C17—C18	1.495 (7)
C5—C6	1.389 (5)	C17—H17A	0.9700
C5—H5	0.9300	C17—H17B	0.9700
C6—C7	1.497 (5)	C18—C19	1.462 (6)
C7—H7A	0.9700	C18—H18A	0.9700
C7—H7B	0.9700	C18—H18B	0.9700
C8—C9	1.383 (4)	C19—C20	1.541 (6)
C8—C13	1.397 (4)	C19—H19A	0.9700
C9—C10	1.382 (6)	C19—H19B	0.9700
C9—H9	0.9300	C20—H20A	0.9700
C10—C11	1.369 (6)	C20—H20B	0.9700
			
C8—Sb—C1	98.17 (12)	C11—C12—C13	119.9 (4)
C8—Sb—N1	77.37 (10)	C11—C12—H12	120.0
C1—Sb—N1	75.86 (11)	C13—C12—H12	120.0
C8—Sb—Cl1	91.80 (8)	C8—C13—C12	119.9 (3)
C1—Sb—Cl1	92.95 (10)	C8—C13—C14	120.4 (3)
N1—Sb—Cl1	162.92 (7)	C12—C13—C14	119.6 (3)
C14—N1—C7	110.3 (3)	N1—C14—C13	112.7 (3)
C14—N1—C15	115.1 (3)	N1—C14—H14A	109.0
C7—N1—C15	110.9 (3)	C13—C14—H14A	109.0
C14—N1—Sb	107.40 (18)	N1—C14—H14B	109.0
C7—N1—Sb	103.79 (19)	C13—C14—H14B	109.0
C15—N1—Sb	108.64 (19)	H14A—C14—H14B	107.8
C2—C1—C6	119.4 (4)	C16—C15—C20	111.2 (3)
C2—C1—Sb	125.8 (3)	C16—C15—N1	114.1 (3)
C6—C1—Sb	114.7 (2)	C20—C15—N1	110.9 (3)
C3—C2—C1	120.4 (4)	C16—C15—H15	106.7
C3—C2—H2	119.8	C20—C15—H15	106.7
C1—C2—H2	119.8	N1—C15—H15	106.7
C4—C3—C2	119.9 (4)	C15—C16—C17	109.6 (4)
C4—C3—H3	120.0	C15—C16—H16A	109.7
C2—C3—H3	120.0	C17—C16—H16A	109.7
C3—C4—C5	120.6 (4)	C15—C16—H16B	109.7
C3—C4—H4	119.7	C17—C16—H16B	109.7
C5—C4—H4	119.7	H16A—C16—H16B	108.2
C4—C5—C6	120.2 (4)	C18—C17—C16	111.0 (4)
C4—C5—H5	119.9	C18—C17—H17A	109.4
C6—C5—H5	119.9	C16—C17—H17A	109.4
C5—C6—C1	119.5 (3)	C18—C17—H17B	109.4
C5—C6—C7	120.4 (3)	C16—C17—H17B	109.4
C1—C6—C7	120.0 (3)	H17A—C17—H17B	108.0
N1—C7—C6	110.1 (3)	C19—C18—C17	111.5 (4)
N1—C7—H7A	109.6	C19—C18—H18A	109.3
C6—C7—H7A	109.6	C17—C18—H18A	109.3
N1—C7—H7B	109.6	C19—C18—H18B	109.3
C6—C7—H7B	109.6	C17—C18—H18B	109.3
H7A—C7—H7B	108.2	H18A—C18—H18B	108.0
C9—C8—C13	118.7 (3)	C18—C19—C20	111.6 (4)
C9—C8—Sb	124.7 (3)	C18—C19—H19A	109.3
C13—C8—Sb	116.3 (2)	C20—C19—H19A	109.3
C10—C9—C8	120.9 (4)	C18—C19—H19B	109.3
C10—C9—H9	119.5	C20—C19—H19B	109.3
C8—C9—H9	119.5	H19A—C19—H19B	108.0
C11—C10—C9	120.0 (4)	C15—C20—C19	109.5 (4)
C11—C10—H10	120.0	C15—C20—H20A	109.8
C9—C10—H10	120.0	C19—C20—H20A	109.8
C10—C11—C12	120.4 (4)	C15—C20—H20B	109.8
C10—C11—H11	119.8	C19—C20—H20B	109.8
C12—C11—H11	119.8	H20A—C20—H20B	108.2
			
C8—Sb—N1—C14	−17.0 (2)	C1—Sb—C8—C13	−68.2 (3)
C1—Sb—N1—C14	84.9 (2)	N1—Sb—C8—C13	5.3 (2)
Cl1—Sb—N1—C14	34.7 (4)	Cl1—Sb—C8—C13	−161.4 (2)
C8—Sb—N1—C7	−133.8 (2)	C13—C8—C9—C10	0.8 (5)
C1—Sb—N1—C7	−31.9 (2)	Sb—C8—C9—C10	175.3 (3)
Cl1—Sb—N1—C7	−82.1 (3)	C8—C9—C10—C11	−1.9 (6)
C8—Sb—N1—C15	108.1 (2)	C9—C10—C11—C12	2.6 (6)
C1—Sb—N1—C15	−150.0 (2)	C10—C11—C12—C13	−2.2 (6)
Cl1—Sb—N1—C15	159.8 (2)	C9—C8—C13—C12	−0.5 (5)
C8—Sb—C1—C2	−91.1 (3)	Sb—C8—C13—C12	−175.4 (3)
N1—Sb—C1—C2	−165.8 (3)	C9—C8—C13—C14	−176.9 (3)
Cl1—Sb—C1—C2	1.2 (3)	Sb—C8—C13—C14	8.2 (4)
C8—Sb—C1—C6	92.5 (2)	C11—C12—C13—C8	1.2 (5)
N1—Sb—C1—C6	17.8 (2)	C11—C12—C13—C14	177.6 (3)
Cl1—Sb—C1—C6	−175.2 (2)	C7—N1—C14—C13	137.9 (3)
C6—C1—C2—C3	−0.9 (6)	C15—N1—C14—C13	−95.7 (3)
Sb—C1—C2—C3	−177.2 (3)	Sb—N1—C14—C13	25.4 (3)
C1—C2—C3—C4	0.1 (7)	C8—C13—C14—N1	−24.4 (4)
C2—C3—C4—C5	0.2 (8)	C12—C13—C14—N1	159.2 (3)
C3—C4—C5—C6	0.3 (7)	C14—N1—C15—C16	−72.1 (4)
C4—C5—C6—C1	−1.0 (6)	C7—N1—C15—C16	54.0 (4)
C4—C5—C6—C7	176.9 (4)	Sb—N1—C15—C16	167.5 (3)
C2—C1—C6—C5	1.3 (5)	C14—N1—C15—C20	54.4 (4)
Sb—C1—C6—C5	178.0 (3)	C7—N1—C15—C20	−179.5 (3)
C2—C1—C6—C7	−176.6 (3)	Sb—N1—C15—C20	−66.0 (3)
Sb—C1—C6—C7	0.0 (4)	C20—C15—C16—C17	56.9 (5)
C14—N1—C7—C6	−74.4 (3)	N1—C15—C16—C17	−176.8 (4)
C15—N1—C7—C6	156.9 (3)	C15—C16—C17—C18	−55.8 (6)
Sb—N1—C7—C6	40.4 (3)	C16—C17—C18—C19	56.2 (7)
C5—C6—C7—N1	151.3 (3)	C17—C18—C19—C20	−57.1 (7)
C1—C6—C7—N1	−30.7 (4)	C16—C15—C20—C19	−57.3 (5)
C1—Sb—C8—C9	117.3 (3)	N1—C15—C20—C19	174.6 (3)
N1—Sb—C8—C9	−169.3 (3)	C18—C19—C20—C15	57.0 (6)
Cl1—Sb—C8—C9	24.0 (3)		

### Hydrogen-bond geometry (Å, °)

**Table:**

*D*—H···*A*	*D*—H	H···*A*	*D*···*A*	*D*—H···*A*
C7—H7A···Cl1^i^	0.97	2.80	3.695 (4)	154.

Symmetry codes: (i) −*x*+1, −*y*+2, −*z*.
